# Multi-length scale bioprinting towards simulating microenvironmental cues

**DOI:** 10.1007/s42242-018-0014-1

**Published:** 2018-05-25

**Authors:** Elisabeth L Gill, Xia Li, Mark A. Birch, Yan Yan Shery Huang

**Affiliations:** 1 Department of Engineering, University of Cambridge, Trumpington Street, Cambridge, CB2 1PZ UK; 2 Division of Trauma and Orthopaedic Surgery, Department of Surgery, University of Cambridge, Cambridge, UK

**Keywords:** 3D bioprinting, Electrospinning, Additive manufacturing, Microenvironment, Disease modelling, Tissue engineering

## Abstract

It is envisaged that the creation of cellular environments at multiple length scales, that recapitulate in vivo bioactive and structural roles, may hold the key to creating functional, complex tissues in the laboratory. This review considers recent advances in biofabrication and bioprinting techniques across different length scales. Particular focus is placed on 3D printing of hydrogels and fabrication of biomaterial fibres that could extend the feature resolution and material functionality of soft tissue constructs. The outlook from this review discusses how one might create and simulate microenvironmental cues in vitro. A fabrication platform that integrates the competencies of different biofabrication technologies is proposed. Such a multi-process, multiscale fabrication strategy may ultimately translate engineering capability into an accessible life sciences toolkit, fulfilling its potential to deliver in vitro disease models and engineered tissue implants.

## Introduction

Advances in 3D bioprinting and biofabrication are accelerating the progress of biological tissue construction with greater complexity and are beginning to realise applications in tissue engineering and in vitro disease modelling [1–5]. Functional tissue formation requires synergistic combination of biologically active materials and bioreactor technology. Hence, tissue assembly often requires configuration of a diverse range of materials and phases, from cell solutions to polymer support structures, at multiple length scales. 3D bioprinting consists of a variety of strategies including those widely used in additive manufacturing mechanisms, such as Inkjet printing [6], microextrusion [7], and stereolithography (SLA) [8–11]. Each printing mechanism has its own associated merits and drawbacks with regard to processing and their subsequent effects on cell behaviour. Combining multiple printing mechanisms in parallel has the potential to unite their respective strengths and create multi-material, hierarchical structures which simulates those of biological tissues.

**Table 1:** Commercially available bioprinters which support multiple printing mechanisms [7, 8, 10]

Company model (origin)	Biofabrication mechanisms	Axis resolution
3D Bioprinting Solutions’ FAB (Russia)	Photocuring,	$5\,\upmu \hbox {m}$
	Electromagnetic pneumatic extrusion	
Advanced Solutions ‘BioAssembly Bot’ (USA)	Pneumatic extrusion	$\sim \,\upmu \hbox {m}$
Aether ‘Aether 1’ (USA)	Pneumatic extrusion	$1\,\upmu \hbox {m}\,(XY)$
	Inkjet/droplet	0.43 nm (Z)
	Filament meltextrusion	
	Photocuring	
Allevi ‘Allevi 6’ (USA)	Pneumatic microextrusion	$<1\,\upmu \hbox {m}$
	Photocuring	(XYZ)
Aspect Biosystems ‘RX1’ (Canada)	Pneumatic extrusion	$\sim \,\upmu \hbox {m}$
Cellink ‘Bio X’ (USA)	Pneumatic extrusion	$1\,\upmu \hbox {m}$
	Filament meltextrusion	(XYZ)
	Mechanical extrusion	
	Inkjet	
	Photocuring	
Cyfuse ‘Regenova’ (Japan)	Spheroid stacking and maturationin needle array	Spheroid diameter
EnvisionTEC Bioplotter	Pneumatic extrusion	$1\,\upmu \hbox {m}$
(German)	Photocuring	(XYZ)
GeSim ‘BioScaffolder 3.2’	Pneumatic extrusion	$2\,\upmu \hbox {m}\,(XY)$
(Germany)	Piezoelectric nanoliter-	$10\,\upmu \hbox {m}\,(Z)$
	pipetting(Inkjet)	
	Melt electrospinning	
	Photocuring	
Organovo’s NovoGen MMX (USA)	Mechanical microextrusion	$20\,\upmu \hbox {m}$
Poietis ‘NGB 17.03’ (France)	LIFT	$20\,\upmu \hbox {m}$
RegenHU’s ‘3D Discovery’ (Switzerland)	Pneumatic extrusion	$5\,\upmu \hbox {m}$
	Mechanical extrusion	
	Inkjet/droplet	
	Photocuring	
	Melt electrospinning	
	Filament melt extrusion	
SunP Biotech International,LLC	Mechanical extrusion	$5\,\upmu \hbox {m}$
‘Alpha-‘ Series(USA/China)	Photocuring	

Many commercially available bioprinters now offer multi-nozzle systems for depositing different materials and further the ability to accommodate different printing mechanisms. With examples shown in Table 1, these printing mechanisms range from the extrusion, inkjet, light curing processes, to melt electrospinning [7, 8, 10]. With the availability of these tools, tissue and scaffold structures can be potentially made with enhanced complexity, at the micro- and potentially nanoscale, and furthermore integrate multiple functional components [12, 13].

In this article, we will draw philosophy from the stem cell microenvironments [14, 15], to consider how different biofabrication techniques can contribute to the spatial patterning of some known microenvironmental cues. Specific focus is placed on extracellular matrices (ECMs) and ECM-like materials. The review starts by considering the structure and function of native ECM and that the analogue of ECM exists in two distinct physical phases, fibril and non-fibrillar (or gel) structures, with separate yet intertwining roles in regulating cellular behaviour. Subsequently, we will evaluate the technology currently available to process the two ECM phases independently. Lastly, we will consider how the integration between printed cells, extracellular matrices, and microfluidics can potentially lead to more precise and flexible recreation of microenvironmental cues in vitro. Cross-comparison between different biofabrication techniques in their operation length scale, materials library and processing merits are provided in the forms of tables and schematics.

**Fig. 1:**
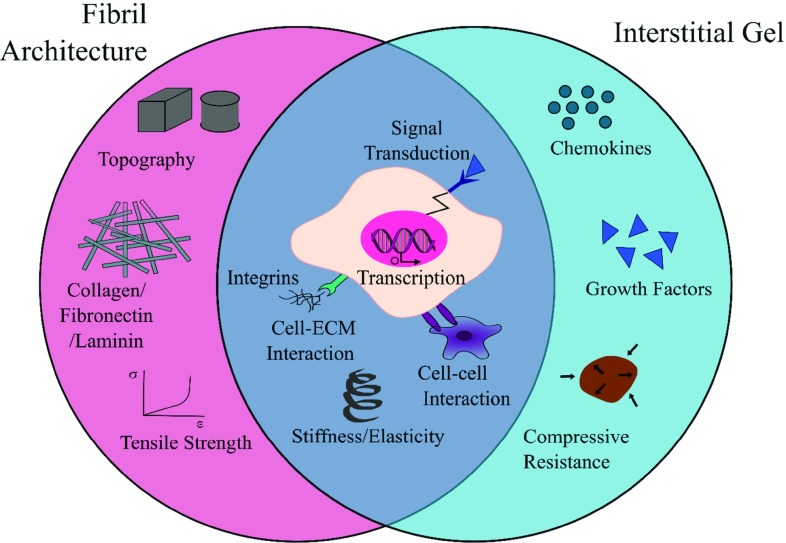
Two physical components of the ECM work in synergy. Contributions from the fibril architecture and interstitial matrix regulate cell function from the bulk tissue to the subcellular level

## Extracellular matrices and their functions

In vivo, the extracellular matrix (ECM) forms part of the cellular microenvironment, providing structural support as well as biochemical and biomechanical cues that regulate tissue differentiation, homeostasis and morphogenesis [16]. It is comprised of polysaccharides, structural proteins, bound growth factors and cytokines, and extracellular vesicles that were secreted and maintained by the cells that once inhabited it [16]. The hierarchical architecture of ECMs have features that span over 7 orders of magnitude, from the sub-nanometre level of molecular sequence, meso-scale fibrils, to the millimetre level of distinct tissue layers [15, 17, 18]. ECMs have complex compositions, are organ-specific and remodel dynamically with age or disease [16–19]. Despite such detailed complexity, an ECM could be seen to structurally exist with two phases, a fibril architecture and a hydrated interstitial gel. For most ECM types, from bone to heart to brain, modelling the ECM as a fibre-reinforced matrix holds true for biomechanical behaviour [20]. The relative abundance and structure of these two components vary drastically in different tissue types from ‘hard’ to ‘soft’ and can simplistically account for the diverse biomechanical properties of bulk tissues. However, the implications of fibril and gel phases extend beyond biomechanical functions. A schematic illustration of the functions of these two phases is shown in Fig. 1. The fibrous network provides tensile strength; it further provides the substrate and topographic cues to regulate cell adhesion, guiding migration and directing tissue formation. ECM components that fall in this category include collagen, fibronectin, elastin and laminin [16]. The interstitial gel consists of a watery mix of proteoglycans, which maintain cell hydration and homeostasis through buffering and binding soluble factors. A good example of its functionality is the hydrated glycoproteins, hyaluronan and proteoglycans present in the cartilage tissue, which interact with the fibril network to provide resistance against excessive tissue compression [16].

While adhesion molecules and topography relate to surface properties of a material, the internal 2D and 3D architecture is an intrinsic contributor to the material’s bulk stiffness. Native soft tissues cover a broad range of apparent bulk moduli from that of the brain (a couple of hundred Pascals) to tendons and cartilage which are in the mega-Pascals range [21]. As cells attach and pull against a matrix, there is an assessment of the substrate’s elastic resistance through a complex array of cell surface and intracellular proteins that combine to form a mechano-transducer that determines how much force is required for matrix deformation [22, 23]. The stiffness of a cell interface therefore greatly influences cell morphology and function and plays a significant part in the extracellular environment. Many studies have attempted to exploit this within the context of stem cells, aiming to design microenvironment combinations to specify cell lineage in a tissue-dependent manner, e.g., [23, 24]. Within native tissues, the composition and structure of the fibril versus non-fibril phases of ECMs determines the tissue stiffness. As alluded to in Fig. 1, high tensile stiffness is a property largely dependent on the collagen, elastin and fibrin fibril architecture that also provides nanotopographic cues in the ECM [16]. Compressive stiffness is normally provided by the charged proteoglycan network (which is considered as an interstitial gel here) [24, 25]. There is the potential to independently tune the two phases to improve tissue microenvironmental mimicry with the various biofabrication techniques developed, as is reviewed subsequently.

**Fig. 2:**
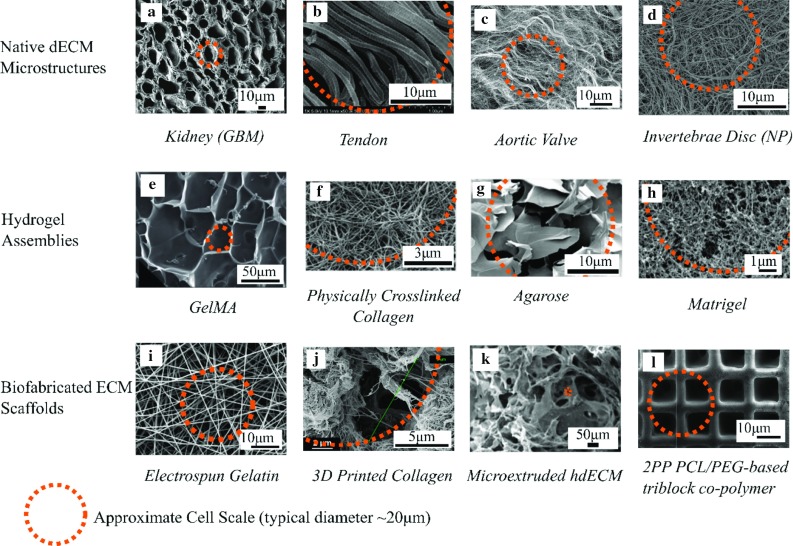
Microstructure comparison between decellularised ECM and biofabricated matrix materials. **a**–**d** SEM images of example dECMs (decellularised extracellular matrices), kidney (glomerular basement membrane) [85] tendon [86] aortic valve [87] and **d** invertebrate disc (nucleus pulposus) tissues [88]. **e**–**h** The microarchitecture of several popular hydrogels, with gelatine methacryloyl (GelMA) [89] collagen [90] agarose [91], and matrigel [92]. **i**–**l** Biofabricated structures, with electrospinning [93] gel extrusion of collagen [94] microextrusion of decellularised ECM [95] and 2PP [96]. The orange outline indicated on each image shows an approximation of the scale of a cell with respect to the structure. Copyright: **a** Copyright© (2013), Elsevier; **b** Copyright© (2011), John Wiley and Sons. **c** under CC BY license. **d** Copyright© (2013), Elsevier. **e** Copyright© (2014) Royal Society of Chemistry. **f** Copright© (2007) Elsevier. **g** Under CC BY license. **h** under CC BY license. **i** Copyright© (2006) Elsevier **j** Copyright © (2018) Springer International Publishing AG. **k** Copyright© (2016) Elsevier **l** Copyright© (2009) American Chemical Society

## Replicating extracellular microenvironment function

To illustrate the microstructures of native ECM, scanning electron microscope (SEM) images of selected decellularised soft tissues are shown in Fig. 2a–d. These images display the diversity and heterogeneity of ECM microstructures, from fibrous to macro-porous characteristics. Total replication of the ECM is an impossible task and may not be necessary. Prioritising the features that recreate some bioinspired functionalities, as is the reasoning in the following paragraphs, may be adequate for many applications.

Hydrogels are commonly used to recapitulate the ECM environment. As indicated in Fig. 2e–h, bioprinted hydrogel assemblies exhibit largely homogenous, isotropic structures at the single cell level. This is distinct to the heterogeneous and often anisotropic fibrous architecture shown in Fig. 2a–d for the native matrices. Thus, whilst selected hydrogel materials may be adequate to model some aspects of ECM features at molecular level, they may lack the ability to present topographical features of a cell interface. In contrast, while synthetic fibrous structures such as those produced by electrospinning (see Fig. 2i) can provide topography and contact guidance, the close packing of conventional electrospun fibre scaffolds can be restrictive to cell infiltration and long-term viability [26–28]. Moving to the multicellular scale, the macro-porosity of the biofabricated structures shown in Fig. 2j , k can be over an order of magnitude greater than the cellular level, which is permissive for cell infiltration and proliferation. It is also important to note that the chosen fabrication technique strongly influences the matrix architecture. As shown in Fig. 2f, j, the same chemical constituent collagen exhibits distinct topography and consequently disparate macroscale scaffold properties dependent on the material processing technique.

**Fig. 3:**
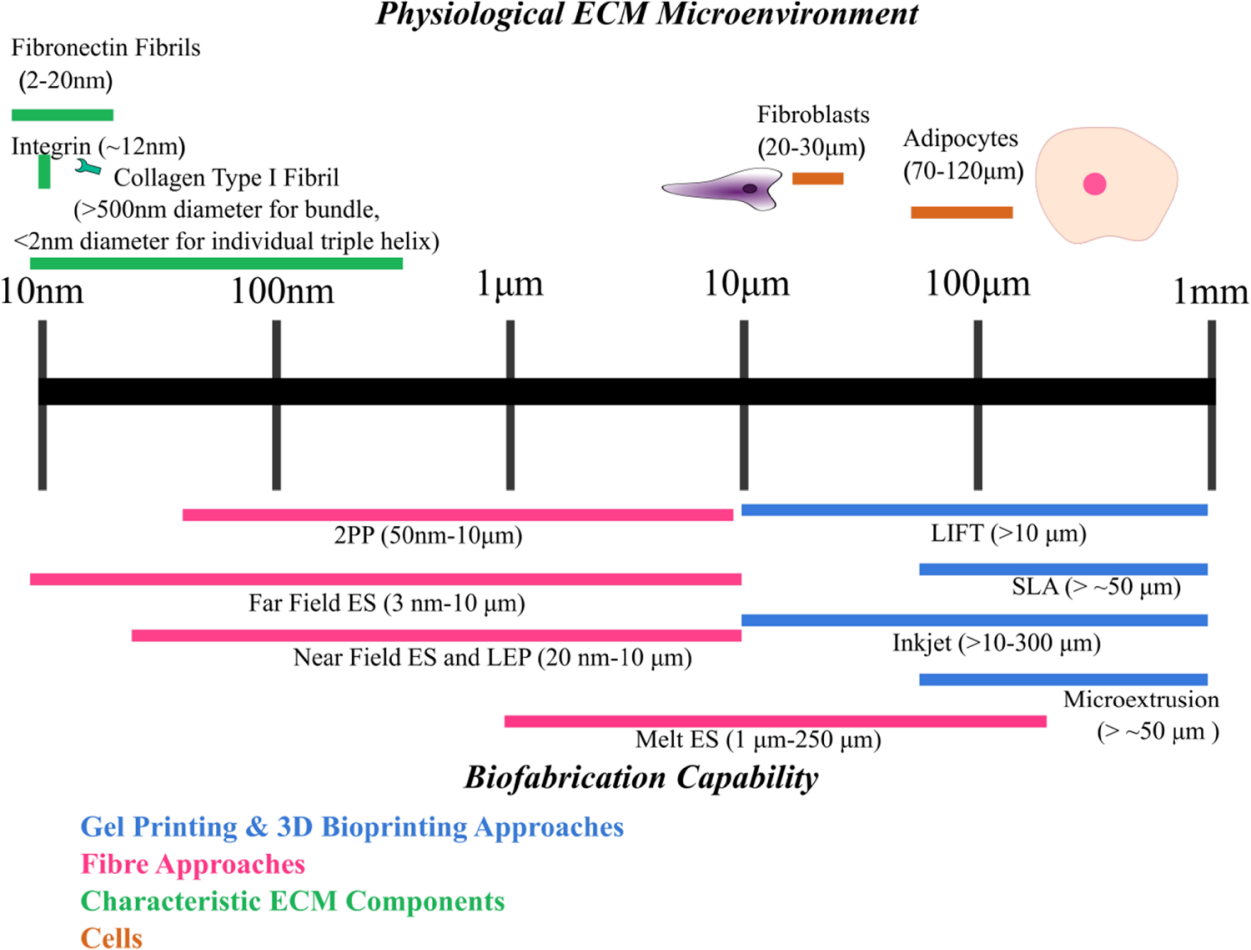
A scale lengths bar contrasting tissue architectural features to the typical resolution attainable from current biofabrication techniques. [10, 11, 49, 97–102] 2PP = two-photon polymerisation; ES = electrospinning; LIFT = laser-induced forward transfer; SLA = stereolithography; LEP = low-voltage electrospinning patterning.

Focusing on the fabrication feature size, Fig. 3 summarises the scale lengths at which the core biofabrication techniques operate. By contrasting these length scales to some of the key features of the ECM, it is shown that cross-length scale biochemical and structural mimicry requires the combination of multiple fabrication techniques. In addition to the restrictions on feature size, the various techniques also have their strengths and limitations due to their processing mechanisms, which underpins their compatibility with cells or heat-sensitive material printing (see Tables 2, 3). For a more in-depth discussion, the following subsections consider the technical details to replicate the two phases of the ECM previously identified: the non-fibril (gel) matrix and fibril architecture. They are reviewed independently according to their intended functionality, starting with methods of designing the gel matrix *via* various means of gel printing and in situ assembly.

**Table 2:** Comparison of biofabrication techniques

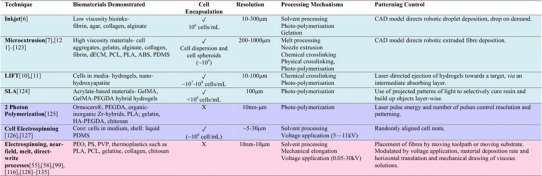	

Blue are techniques suited for gel printing, pink fibril printing, and purple uncategorised *ABS* acrylonitrile–butadiene–styrene; *dECM* decellularised extracellular matrix; *GelMA* gelatine methacryloyl; *HA* hyaluronic acid; *NFES* near-field electrospinning; *PCL* polycaprolactone; *PDMS* polydimethylsiloxane; *PEGDA* poly(ethylene glycol) diacrylate; *PEO* poly(ethylene oxide); *PLA* polylactic acid; *PS* polystyrene; *PVP* polyvinylpyrrolidone

### Gel matrix assembly

The precise and ‘safe’ patterning of cells and biomolecules can define key microenvironmental factors such as cell–cell interactions and cell signalling. In most cases, 3D bioprinting utilises a hydrogel matrix to encapsulate cells or biomolecules (the combination of which is commonly termed bioink). The primary function of the hydrogel is to deliver living components in a hydrated environment, immobilise them in their designated position with gelling mechanisms and protect them from the processing conditions [29]. The key exceptions are ‘scaffold-free’ tissue spheroid printing approaches [30, 31], which applies developmental biology and tissue morphogenesis. In the following, we will briefly overview the design of bioinks in terms of the attainable shape fidelity, printing resolution and molecular biomimicry with current technologies. Bioink material properties and their influence on cell viability has already been reviewed in work such as that of the Shah and co-workers [32]. Here, we instead focus on how these printed bioink or gel characteristics will contribute to the multi-process integration.

*Shape fidelity and print resolution* The printing of cell-laden hydrogels in 3D often presents a dichotomy between maintaining built up spatial arrangement, preserving delicate material properties and uninhibited future cell functionality. To attain optimal shape fidelity, high polymer concentrations are necessary which may limit cell viability post-printing [33, 34] as cell proliferation and infiltration into the scaffold is hindered. On the other hand, rheology, hydrogel cross-linking mechanisms, surface tension, and liquid–surface interactions determine the resolution of printed hydrogels and soft materials [10, 35, 36]. Hence, the resolution of the printed constructs will be significantly lower than the described axes resolution of commercially available bioprinters (as stated in Table 1). These factors have established a ‘biofabrication window’ referring to the conflict which needs to be reconciled between the mechanical demands for printed shape fidelity and establishing physiologically relevant stiffness and substrate cues for cell function [35]. Additional limitations on printed resolution are related to the inherent restriction on nozzle size to avoid cell membrane damage due to shear stress or issues of nozzle clogging. The rheology of the bioink can be highly influential to mitigate these issues [10, 37, 38]. A common strategy is to design bioink chemistry to have shear-thinning behaviour [39], which enables the bioink to have lower viscosity during nozzle extrusion to reduce cell damage, and yet maintain print resolution through the increased viscosity immediately following deposition.

*Hydrogel chemistry* Hydrogel cross-linking is necessary to fix printed gels in place and determines their mechanical behaviour. Naturally derived hydrogels usually use physical cross-linking mechanisms which are either ionic or temperature dependent [35]. For example, naturally derived collagen gel assembles upon physical cross-linking at the point of pH neutralisation at physiological temperature. Consequently, it is widely used in the field of biology. However, physical cross-linking mechanisms generally produce mechanically weak gels that often have slow cross-linking dynamics, which are difficult to control. Compared to physical cross-linking, photoinitiated chemical polymerisation offers rapid cross-linking with controllable reaction dynamics, which allows greater adjustment of cross-link density and can produce higher-resolution macrostructures. Synthetically derived poly ethylene glycol (PEG)-based hydrogels have been particularly favoured due to the ease with which their elastic and degradation properties can be tailored [40, 41]. Much research has been conducted in the effort to find optimal photoinitiators and refine their concentration in order to minimise radical-induced damage to the polymer backbone and to cells [42–44]. Chemical modification of naturally derived biomaterials to facilitate photopolymerisation has also attracted significant interest. For example, photosensitive gelatin methacryloyl (GelMA) hydrogel is especially popular [45, 46]. Aside from controlling the built-shape, hydrogel cross-links also strongly influences whether the resident cells can perform matrix remodelling and matrix degradation. Recent developments in supramolecular assembly hydrogels can be used to tailor reversible hydrogel bonding. This may yield structures with good temporary mechanical properties upon deposition, which later have more appropriate permeability and degradation properties for tissue maturation [47, 48].

*Two-photon polymerisation (2PP)* high-resolution printing achieved by 2PP can be imparted by the strong covalent bonds that take place between inorganic and organic polymer components, which enable structures to withstand greater stresses post-fabrication [49]. Improved printing resolution is often a result of enhanced mechanical stability given by a high degree of polymerisation, utilising higher strength intermolecular bonding. The correlation of stiffness with bond strength varies between polymerisation methods. In some instances, the strength of these bonds yields elastic properties ($\sim$ 7.2  GPa for ORMOCER ceramic [50]) that are alien to that of soft tissues ($\sim$ 1 kPa–100 MPa) [51, 52].

### 2D and 3D fibre patterning

As introduced in Sect. 2, the fibrous structures that exist in the ECM and their interplay with cells provide critical mechanical, topographic and substrate environmental cues to cell function. Hydrogel-based 3D printed structures cannot reach the resolution of the native meso-fibril architecture; see Fig. 2, which is considered a solid structure with diameters from tens of nanometres to a few microns. As previously discussed, the resolution constraints are not due to precision in robotic control, but the stabilising mechanisms in biomaterials processing. Electrospinning can produce fibres which offer topographical function but conventionally the technique has poor patterning control, particularly in 3D. Electrohydrodynamic fibre writing techniques, such as melt electrospinning and near-field electrospinning, can potentially address these limitations in a manner akin to additive layer manufacture. Incorporating designable fibril networks within a cellular interface is a logical step towards improving the functionality of bioprinted tissues, which to date is predominated by hydrogel printing. In the following subsection, we summarise advances in 3D nanofibril patterning and approaches suitable for process integration with 3D bioprinting.

The high voltage applied in conventional electrospinning (10–30 kV) is utilised to draw fine fibres but it is also responsible for poor fibre patterning control. Melt processing will give better controllability in patterning precision due to the melt elastic property and by eliminating of the influence of solvent evaporation [53]. Melt electrospinning has already been incorporated in conjunction with other more well-established printing mechanisms such as inkjet and extrusion on some commercial bioprinting systems. Conductive patterned substrates can also act as effective guides to the deposition path of electrospun fibres, though are very sensitive to disturbance in local substrate electric field. This strategy has shown to produce layered placement of melt electrospun fibres, as reported by Brown et al. [53, 54]. However, the high-temperature melt processing and high applied voltage (> 5 kV) utilised by the melt electrospinning processing confines material selection to thermoplastics, further limiting the fibre surface properties and biofunctionality of the fibril network.

An electrostatically driven, solution-based approach will naturally enable processing of a wider range of materials compared to a mechanically driven technique (e.g., liquid drawing). Near-field electrospinning (NFES) techniques lower the applied voltage (1–5 kV range) but achieve the same tensile drawing effect by reducing the tip-collector distance to maintain the electric field strength [55]. Rapid linear movement of either collecting substrate or the printhead can create micron to sub-micron-level fibres. This compensates for the absence of the fibre elongation from the bending instability and facilitates fibre patterning in 2D. Solvent processing also offers the potential to manipulate fibre cross section and adjoining behaviour; a recent study utilised the merging behaviour of wet fibres to form strong orthogonal fibre junctions. The interconnected fibre network possessed enhanced mechanical properties and demonstrated a collagen and PCL blend thus showing prospective applications in mechanical reinforcement of natural biomaterials [56]. By tuning the substrate properties, it is possible to build 3D structures using the fibre patterns [57]. Recent development in the near-field techniques has further lowered the operating voltage to the range of 100 V. Low-voltage electrospinning patterning (LEP) utilises both mechanical and electrical forces for fibre initiation, in addition to the mechanical stretching of the fibre against a collection substrate. As such, deposition of suspended biological fibres was demonstrated on 3D printed supports, in addition to suspended fibre membranes over microfluidic channels [58]. The main benefit of lowering the applied voltage in electrostatic processing is the minimised damage to the bioactive components, and greater flexibility to combine with bioprinting mechanisms. Advances in 3D electrospun architecture and solution drawn fibres indicate that with precise tuning of working parameters, complex micro- or even nanoscale structures can foreseeably pattern synthetic and naturally derived biopolymers in 3D.

**Table 3:** Merits and precautions of processing mechanisms commonly used in biofabrication.

Processing mechanism	Merits and associated advantages	Limitations and precautions
Solvent processing	Takes place in ambient conditions.	Residual solvents (if non-biocompatible) could influence cell behaviour
	If solvents are water-based can be helpful for cell hydration	
Physical cross-linking	Selected processes occur under physiological pH and temperature	Weak gelation
		Poor mechanical properties
Chemical cross-linking	Improved control for shape fidelity	Control of cross-linking homogeneity important
	Rapid gelation	Choice of cross-linking agent and amount important to avoid cytotoxicity
Photopolymerisation	Good shape fidelity	Photoirradiation damage to polymer backbone produces free radicals, which can be damaging to cells and degrades biomolecules
	Rapid gelation	Choice of cross-linking agent and amount important to avoid cytotoxicity
Melt processing	No harmful solvent residues	High processing temperatures may be unsuitable to integrate with parallel processing of cells, proteins and some biomaterials
	Control of solidification with temperature	
Voltage application	Improved resolution by overcoming liquid surface tension	Applied currents may affect cell viability
	Can be used as an indirect control of fibre suspension	If solvent is used, need to incorporate adequate solvent removal procedure
		Residual charges may limit patterning capability
Nozzle extrusion	Simple configuration	Shear stresses may lead to cell death or a change in cell phenotype
	Can tune ink rheology properties to incorporate different print functionalities	Limited to ink viscosity greater than 30 mPa/s [10]

### Biphasic fibre–gel architecture

With the complementary roles played by fibres and interstitial gels in a native ECM, one can anticipate that the combination of these two phases could be desired for creating more complex bioinspired structures. Due to current technological limitations in combining these two phases, most studies to date report multistage, manual manipulation. Reviews by, e.g., Bosworth [59], Butcher [60] and more recently Xu [61] et al. detail different methodologies to assemble composite structures. The methodologies to create the biphasic structures can be classified under the categories of lamination, encapsulation, injectable hydrogels, and dual electrospinning [59]. More recently, Fattahi et al. demonstrated a near-field electrospinning process capable of depositing PMMA fibres with controlled alignment layered on top of slabs of collagen hydrogel [62]. So far, the aspirational applications for these composite structures have been motivated by their enhanced biomechanical properties, such as the repair of bone [63, 64], cartilage [65–67], tendons [68], and heart valves [69, 70].

From comparison to either purely hydrogel-based or fibrous scaffolds, existing fibre-reinforced composites have reported enhanced control of cell distribution, viability, and activity afforded by the contact guidance of the fibre components coupled to the tailored permeability with the gel matrix [27, 70, 71]. For example, Han et al. intentionally exploited the small pore size afforded by electrospun fibres to pace the release of neurotrophins from their hydrogel for PC12 neural stem cell differentiation [72]. Xu et al. reported a fibre–hydrogel composite designed to grow cortical bone tissue, the scaffold’s degradation coincided with when stromal bone marrow cells began to secrete their own ECM, followed by mineralisation [64]. Similarly, composites designed to form cartilage-like tissues showed enhanced production of type II collagen and GAGs by chondrocytes [65, 73] and neural progenitor stem cells embedded in a composite showed physiologically relevant gene upregulation [74] when contrasted to single phase gel counterparts.

Finally, microfluidic cell culture has also been coupled with nanofibrous structures as a platform to potentially biomimic the cell microenvironment through control of soluble, mechanical and topographical cues. Wallin et al. integrated nanofibres in various orientations and alignments within a microfluidic chip to mimic the surface topography of the extracellular matrix of glial cells. Simultaneously, a physiologically relevant neurotropic chemical gradient was established in the microfluidic channel to mimic the microenvironmental conditions to model axon outgrowth [75]. This configuration was used to study the interdependency of topographical and chemical cues and their relative strength when acting in alignment and in opposition to one another. The idea of combined modelling of substrate and soluble cues of the ECM can foreseeably be extended to capture greater microenvironmental complexity and extend physiologically relevant in vitro functionality.

**Fig. 4:**
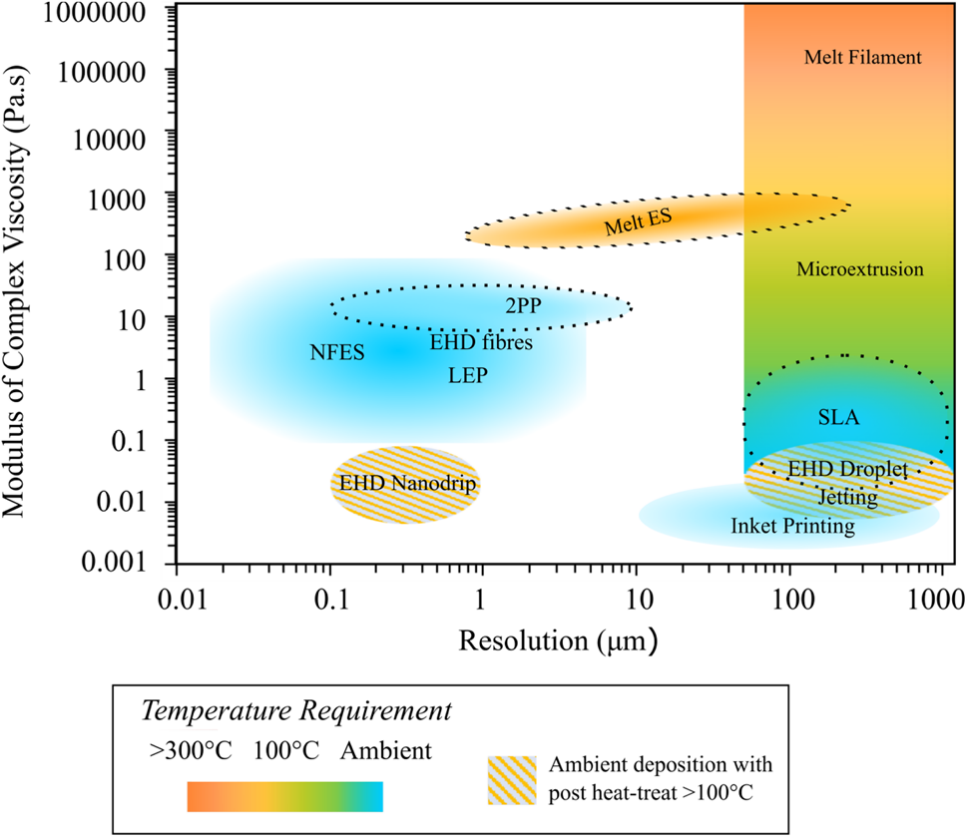
The range of resolution and printing ink or resin rheological properties that different printing mechanisms can operate. The modulus of complex viscosity is used as a generic indicator for the viscoelastic property [9, 13, 78, 103–119] EHD = electrohydrodynamic deposition [120]

## Prospective fabrication of cellular microenvironments at multiscale

With the potential of multi-material processing in an integrated platform, Fig. 4 maps the regions of material viscosity and printed construct resolution that different printing mechanisms and microfabrication technologies operate. By a ‘mix-and-match’ approach towards complementary techniques, one can potentially design a bioinspired cell scaffold offering both biological functions and sensing functions [76]. To attain biological functionality, one should include appropriate microenvironmental cues within the design to influence the living components and be mindful of what cues are created by the fabrication process itself. This warrants the question of whether it will be ultimately possible to position cells in way that maintains macroscale shape fidelity, but does not adversely interfere with microenvironmental cues. Answers may lie in the design of temporary external support structures to allow in situ self-assembly into mature and mechanically stable tissue constructs [77]. In situ bioprinting of small-scale tissue scaffolds could also be key in the development of implantable tissues.

**Fig. 5:**
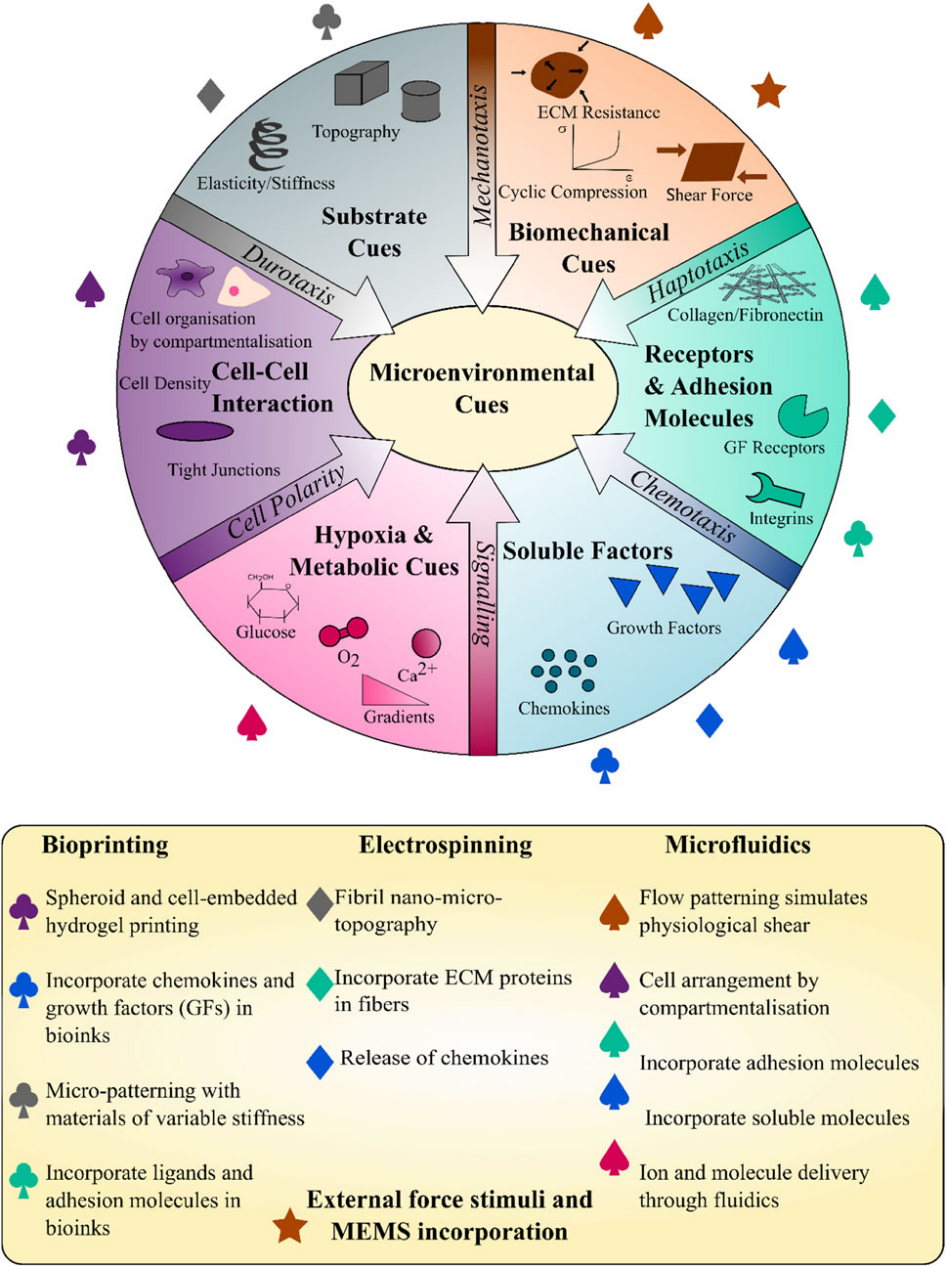
Scheme showing microenvironmental cues [84] and suggested biofabrication techniques suitable to replicate them. Adapted with copyright permission CC BY 4.0.

To illustrate further how a stem cell *niche* concept can potentially be used to guide a biofabrication strategy, Fig. 5 illustrates some of the key microenvironmental cues and indicates potentially suited technique(s) which have the potential to replicate them. Bioink printing (or 3D Bioprinting as illustrated in the figure) is an effective way of placing cell suspensions with adequate precision. Bioink printing can provide versatile and potentially temporary support structures to maintain macroscale shape fidelity. Electrospinning and other fibre drawing technologies can offer a means of creating micro- and nanoscale fibres. In 2D, these have already shown promise for the application of cellular assays. One example used patterned fibril arrays to study endothelial response to ROCK inhibition and this in vitro platform permitted statistical single-cell image cytometry using conventional microscopy [78]. In 3D, the patterning of ECM-like fibres could provide the substrate and topographical cues that soft 3D printed hydrogels cannot offer. Furthermore, they can act to modulate tissue stiffness and other biomechanical properties without compromising cell proliferation and motility. It may also be possible with core–shell electrospun fibres to programme controlled release of soluble factors from within a biodegradable sheath to sustain cell development over a longer period. Microfluidic devices offer the possibility to design chemotactic stimuli and dynamic biomechanical conditions [79–81]. This can be utilised to establish physiologically relevant metabolic cues such as oxygen and ion gradients in addition to perfusing media and soluble factors around the chambers, in the fashion of a miniaturised bioreactor. The shear stresses that ensue from dispersing fluids around cells equally contribute to the biomechanical microenvironmental cues that direct cell fate. Fluidic chambers can also be adapted to co-culturing different cell types in 3D and simulating physiologically relevant mechanical strain by incorporating other microelectromechanical systems (MEMS) features.

Emphasis should be focused on the dynamic aspect of microenvironmental cues that make up a cell *niche* and simulate their naturally integrated multiple feedback mechanisms. As such any foreseeable design should incorporate an element of timing and hence the terminological transition to ‘4D printing.’ This incorporates cues which enable printed objects to continuously evolve under environmental stimuli [82, 83]. Tissue maturation itself can be considered a dynamic ‘4D’ process. Thus, the placement of relevant growth factors and chemokines within hydrogel matrices has the capability to guide cell growth and differentiation post-printing, which is a clear example of how time co-ordination shall need to play a role in future biofabrication strategies.

With tailored design of microenvironmental cues which culture progenitor cell populations, it should be possible to model tissue-specific and even patient-specific responses to environmental stimuli [18]. It will be important to translate the technical advances in biofabrication technology into tools with the designated end user in mind, the biomedical community [84]. Tissue biofabrication will need to be reproducible, validated and economically viable. It is also crucial to ensure that multi-process printing and patterning steps are compatible with one another as well as to allow tissue constructs to support further biochemical analysis, or be appropriately prepared for later in vivo implantation. Continued developments in biofabrication and stem cell technologies have tremendous potential in the intermediate term to revolutionise drug-screening procedures. In the longer term, they have the potential to help advance our fundamental understanding of pathology significantly and ultimately deliver patient-specific clinical treatments and prevention.
